# Associations between sensory processing and electrophysiological and neurochemical measures in children with ASD: an EEG-MRS study

**DOI:** 10.1186/s11689-020-09351-0

**Published:** 2021-01-06

**Authors:** Sarah Pierce, Girija Kadlaskar, David A. Edmondson, Rebecca McNally Keehn, Ulrike Dydak, Brandon Keehn

**Affiliations:** 1 Department of Psychological Sciences, Purdue University, West Lafayette, IN USA; 2 Department of Speech, Language, and Hearing Sciences, Purdue University, West Lafayette, IN USA; 3 Cincinnati Children’s Hospital Medical Center, Imaging Research Center, Cincinnati, OH USA; 4 Department of Pediatrics, Indiana University School of Medicine, Indianapolis, IN USA; 5 School of Health Sciences, Purdue University, West Lafayette, IN USA; 6 Department of Radiology and Imaging Sciences, Indiana University School of Medicine, Indianapolis, IN USA

**Keywords:** Autism spectrum disorder, MRS, EEG, Alpha power, GABA, Glutamate, Sensory processing

## Abstract

**Background:**

Autism spectrum disorder (ASD) is associated with hyper- and/or hypo-sensitivity to sensory input. Spontaneous alpha power, which plays an important role in shaping responsivity to sensory information, is reduced across the lifespan in individuals with ASD. Furthermore, an excitatory/inhibitory imbalance has also been linked to sensory dysfunction in ASD and has been hypothesized to underlie atypical patterns of spontaneous brain activity. The present study examined whether resting-state alpha power differed in children with ASD as compared to TD children, and investigated the relationships between alpha levels, concentrations of excitatory and inhibitory neurotransmitters, and atypical sensory processing in ASD.

**Methods:**

Participants included thirty-one children and adolescents with ASD and thirty-one age- and IQ-matched typically developing (TD) participants. Resting-state electroencephalography (EEG) was used to obtain measures of alpha power. A subset of participants (ASD = 16; TD = 16) also completed a magnetic resonance spectroscopy (MRS) protocol in order to measure concentrations of excitatory (glutamate + glutamine; Glx) and inhibitory (GABA) neurotransmitters.

**Results:**

Children with ASD evidenced significantly decreased resting alpha power compared to their TD peers. MRS estimates of GABA and Glx did not differ between groups with the exception of Glx in the temporal-parietal junction. Inter-individual differences in alpha power within the ASD group were not associated with region-specific concentrations of GABA or Glx, nor were they associated with sensory processing differences. However, atypically decreased Glx was associated with increased sensory impairment in children with ASD.

**Conclusions:**

Although we replicated prior reports of decreased alpha power in ASD, atypically reduced alpha was not related to neurochemical differences or sensory symptoms in ASD. Instead, reduced Glx in the temporal-parietal cortex was associated with greater hyper-sensitivity in ASD. Together, these findings may provide insight into the neural underpinnings of sensory processing differences present in ASD.

**Supplementary Information:**

The online version contains supplementary material available at 10.1186/s11689-020-09351-0.

## Introduction

Autism spectrum disorder (ASD) is an etiologically complex, heterogeneous condition affecting 1 in 54 children, making it one of the most prevalent neurodevelopmental disorders [1]. ASD is a behaviorally defined disorder that is diagnosed on the basis of impairments in social communication and the presence of restricted and repetitive behaviors, including atypical responsivity to sensory stimuli [2]. These differences include hyper-, hypo-, and a mixed pattern of hypo- and hyper-sensitivity to sensory input [3]. Importantly, prior research has shown atypical sensory responsivity is present within the first year of life in high-risk infants later diagnosed with ASD [4], and is associated with other core ASD symptoms, including sociocommunicative impairments [5] and restricted and repetitive behaviors [4]. Thus, insight into the source(s) of sensory processing differences may also assist in the explaining the emergence of the heterogeneous ASD phenotype.

Behavioral responses to incoming sensory information are determined, in part, by one’s cortical state [6]. Electroencephalogram (EEG) is a powerful tool for studying spontaneous brain activation associated with patterns of cortical synaptic activity. Neural oscillations, as measured by EEG, play a key role in brain function and are associated with a variety of perceptual and cognitive processes [7]. In particular, the alpha band (8–12 Hz) has been linked to attentional and perceptual processing [8, 9]. For example, pre-stimulus (i.e., spontaneous) alpha power is associated with the detection of briefly presented visual and tactile stimuli [10–12]. In light of these and other findings, several theories have outlined how alpha activity may play an active role in modulating sensory input [9, 13, 14]. In particular, Jensen and Mazaheri [13] and Mathewson and colleagues [14] have proposed that alpha oscillations may function as a sensory gating mechanism through pulsed inhibition, which is mediated by activity of GABAergic inhibitory interneurons. Together, these theories suggest that ongoing alpha oscillatory activity plays a critical role in shaping perception of and responses to incoming sensory information.

Previous studies have focused on neurophysiological differences in ASD, and how they may contribute to the behavioral characteristics associated with the disorder (see [15], for review). Specifically within the alpha band, reductions in alpha are present as early as 3 [16] to 6 months [17] in infants at high risk for ASD. Further, significantly reduced alpha power has also been shown in school-aged children and adolescents [18–21] as well as adults [22] with ASD (although see [23–25], for evidence of greater, or, [26], for evidence of equivalent alpha power in ASD). Within ASD, differences in alpha power have been related to sensory hypo-responsiveness [27], sensory seeking behavior [28], and greater attention to detail [24], suggesting that they may contribute to the sensory processing differences present in individuals with ASD (see [29], for review).

Based, in part, on evidence of reduced alpha power in ASD, Wang and colleagues [15] suggested that individuals with ASD may display a U-shaped profile of EEG power differences with greater low- (delta, theta) and high- (beta, gamma) and reduced mid-frequency (alpha) power in ASD. They hypothesize that this pattern of ASD-related power differences may reflect atypical patterns of excitatory (glutamate) and/or inhibitory (GABA; E/I) neurotransmitters, which is consistent with models that have proposed that ASD may result from atypically increased cortical excitation (Hussman [30]; Rubenstein and Merzenich [31]). Although in vivo measurement of GABA and glutamate concentrations using magnetic resonance spectroscopy (MRS) has provided mixed results (see [32], for review), region-specific differences in GABA concentrations in ASD have been linked to sensory processing differences [33–35]. However, it is currently unclear whether differences in regional patterns of neurotransmitter concentrations are associated with atypical oscillary activity in ASD, and whether inter-individual differences in alpha power and E/I measures may be related to hyper- and/or hypo-sensory sensitivity in ASD.

The current study examines whether differences in alpha-band power are present in children and adolescents with ASD compared to their TD peers and investigates whether a relationship exists between alpha and measures of ASD symptomatology, specifically sensory processing differences. We hypothesize that children with ASD will exhibit reduced alpha levels compared to their TD peers and that decreased alpha power will be associated with increased sensory processing symptoms in ASD. Furthermore, based on the theoretical and empirical links between excitatory/inhibitory (im)balance and atypical neural oscillations and sensory function in ASD, we also sought to examine the associations between in vivo measures of excitatory and inhibitory neurotransmitters, alpha power, and sensory symptoms. By comparing not only the physiological features of both groups, but mapping those onto measures of ASD symptomatology, findings from the current study may provide a better understanding about the heterogeneous nature of ASD symptoms and their neurophysiological bases.

## Methods

### Participants

Participants included 31 children with ASD and 31 age- and non-verbal IQ-matched TD children (see Table 1). IQ scores were determined based on the Wechsler Abbreviated Scale of Intelligence. Second Edition (WASI-II [36]) or the Differential Abilities Scales, Second Edition (DAS-2 [37]). Clinical diagnoses were confirmed using the Autism Diagnostic Observation Schedule, Second Edition (ADOS-2 [38]), Social Communication Questionnaire (SCQ [39]), and expert clinical judgment according to DSM-5 criteria [2]. Participants in the ASD group were excluded if they had any known non-idiopathic forms of ASD, such as Fragile X syndrome. Children with non-verbal IQ scores < 70 were excluded. Typically developing children and adolescents had no significant ASD symptomatology or family history of ASD as confirmed via parent report.

**Table 1:** Participant characteristics

	ASD	TD	*t* value	*p* value
*N* (male to female)	31 (25:6)	31 (22:9)	-	-
Age (years)	11.3 (1.6); 6.5–14.6	10.6 (1.9); 6.6–15.0	1.48	.14
Verbal IQ	101 (19); 67–154	108 (12); 89–129	− 1.76	.08
Nonverbal IQ	104 (17); 70–136	109 (13); 87–132	− 1.21	.23
SP-2 Registration	53 (20); 18–93	23 (9); 2–41	7.77	< .001
SP-2 Sensitivity	50 (14); 27–86	22 (9); 0–47	9.22	< .001
SP-2 Avoiding	60 (17); 19–96	26 (11); 10–70	9.55	< .001
SP-2 Seeking	42 (17); 14–91	21 (9); 2–52	6.15	< .001

*SP-2* Sensory Profile-2

### Electroencephalography (EEG)

#### EEG acquisition

Participants were instructed to relax, remain as still as possible, and look ahead at a black fixation cross on a gray background. EEG data were recorded for 3, 2-min blocks in this eyes-open resting state. EEG was acquired using 128-channel high-density Geodesic sensor nets (Electrical Geodesics, Inc.; Eugene, OR) with a NetAmps 400 high-input amplifier. Data were collected from 124 of 128 possible channel locations. In order to decrease attrition, EOG electrodes (electrodes placed on the face) were not used. Data were sampled at 500 Hz and referenced to the vertex electrode.

#### EEG analysis

**Fig. 1:**
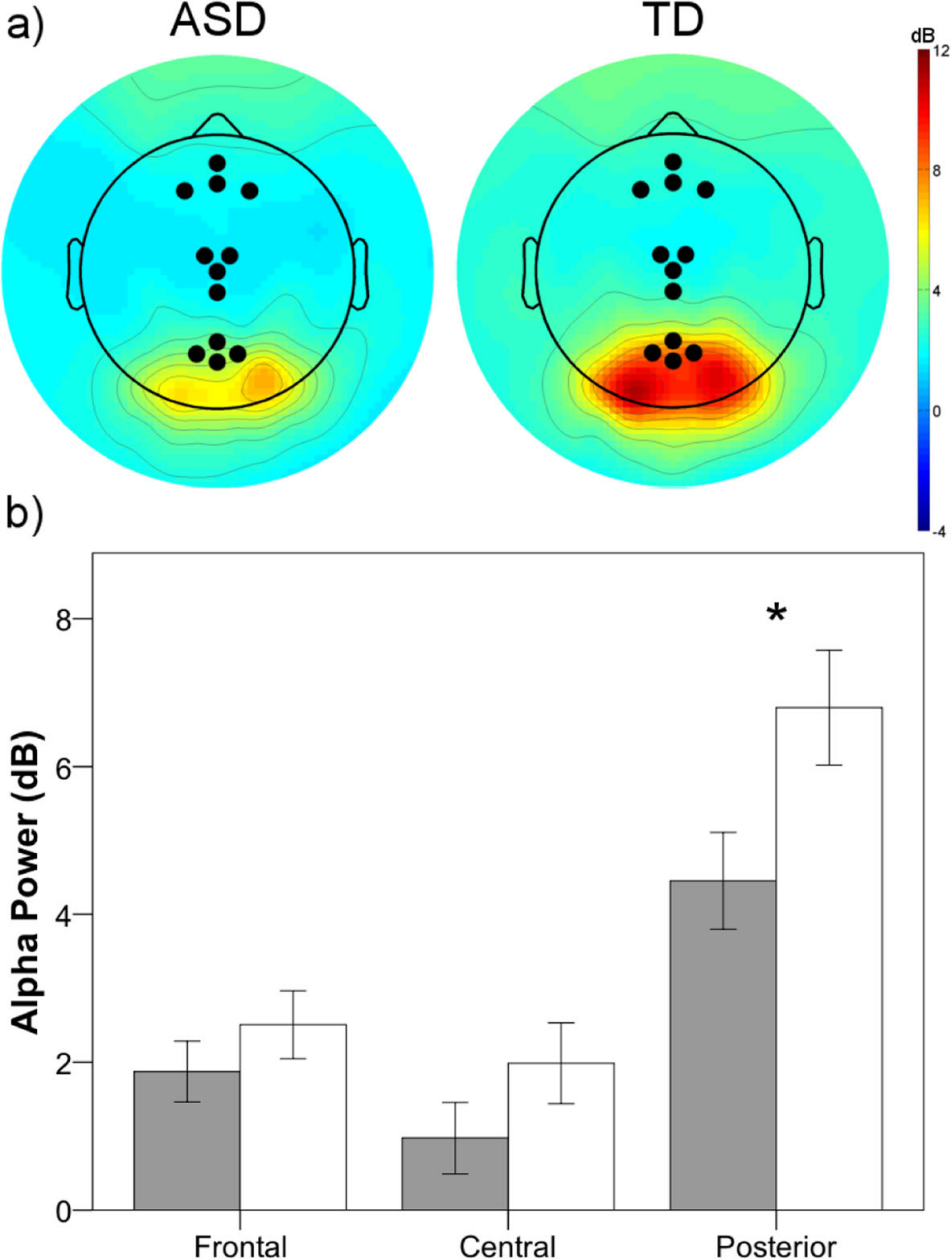
**a** Scalp maps of resting-state alpha (8–12 Hz) power for the autism spectrum disorder (ASD) and typically developing (TD) groups. Regions of interest (ROIs) examined are displayed as black dots. **b** Mean alpha power (db) for ASD (gray) and TD (white) for each ROI. Error bars represent ± 1 SEM. *p < .05

EEG data were processed using MATLAB-based toolbox EEGLAB [40]. Data were filtered (1–50 Hz), bad channels were removed, non-stereotyped artifacts were manually rejected, and then independent component analysis (ICA) completed. Next, SemiAutomatic Selection of Independent Components for Artifact correction in the EEG (SASICA [41]) was used to identify artifacts associated with blinks, saccades, muscle contractions, and bad channels [41]. After removing EEG activity associated with artifactual components, bad channels were replaced using spherical interpolation, and data were re-referenced to the average reference. Groups did not differ significantly in the number of bad channels replaced (ASD: 2 [6]; TD: 1 [2]), *t*(60) = 1.4, *p* = .156, or components removed (ASD: 9 [3]; TD: 8 [3]), *t*(60) = 1.4, *p* = .168. Artifact-corrected data were segmented into 1-s epochs, and epochs containing residual artifacts were rejected. Alpha power (8–12 Hz), expressed as decibels (dB), was extracted from three regions of interest (ROI) each of which consisted of 4 locations across midline frontal (Fz; 4, 11, 16, 19), central (Cz; 7, 55, 106, 129), and posterior electrode locations (Pz; 62, 67, 72, 77; see Fig. 1) using the EEGLAB function spectopo, which computes power spectral density with the frequency resolution of 0.25 Hz.

### Magnetic resonance spectroscopy (MRS)

A subset of age- and IQ-matched participants (ASD = 16; TD = 16) completed the imaging protocol (see Supplementary Table 1). These data have previously been reported in Edmondson et al. [42]. MRI and MRS data were acquired using a 3-T Prisma Siemens scanner with a 64-channel head coil. To minimize movement, participants’ heads were stabilized with foam padding and they were instructed to remain as still as possible and watch a video of their choice for the duration of the scan. A high-resolution T1-weighted image (MPRAGE, TE = 4.91, TR = 2000, TI = 977, FA = 9) was taken for MRS voxel placement and tissue segmentation. Gamma-aminobutyric acid (GABA) was acquired using MEGA-semi-LASER localization (TE = 68, TR = 2000, averages = 128, acquisition time = 8:56) [43]. To measure Glx (glutamate + glutamine) 1H-MR spectroscopy was acquired using semi-LASER localization (TE = 35, TR = 2000, averages = 64, acquisition time = 2:28) [44]. Unsuppressed water acquisitions were acquired as reference scans both for phase and eddy-current corrections as well as for quantification (ratio of metabolite over water) for both semi-LASER and MEGA-sLASER. Parameters for the reference scans were the same as for water suppressed scans with the exception of only 8 averages acquired. Total acquisition time was approximately 45 min.

**Fig. 2:**
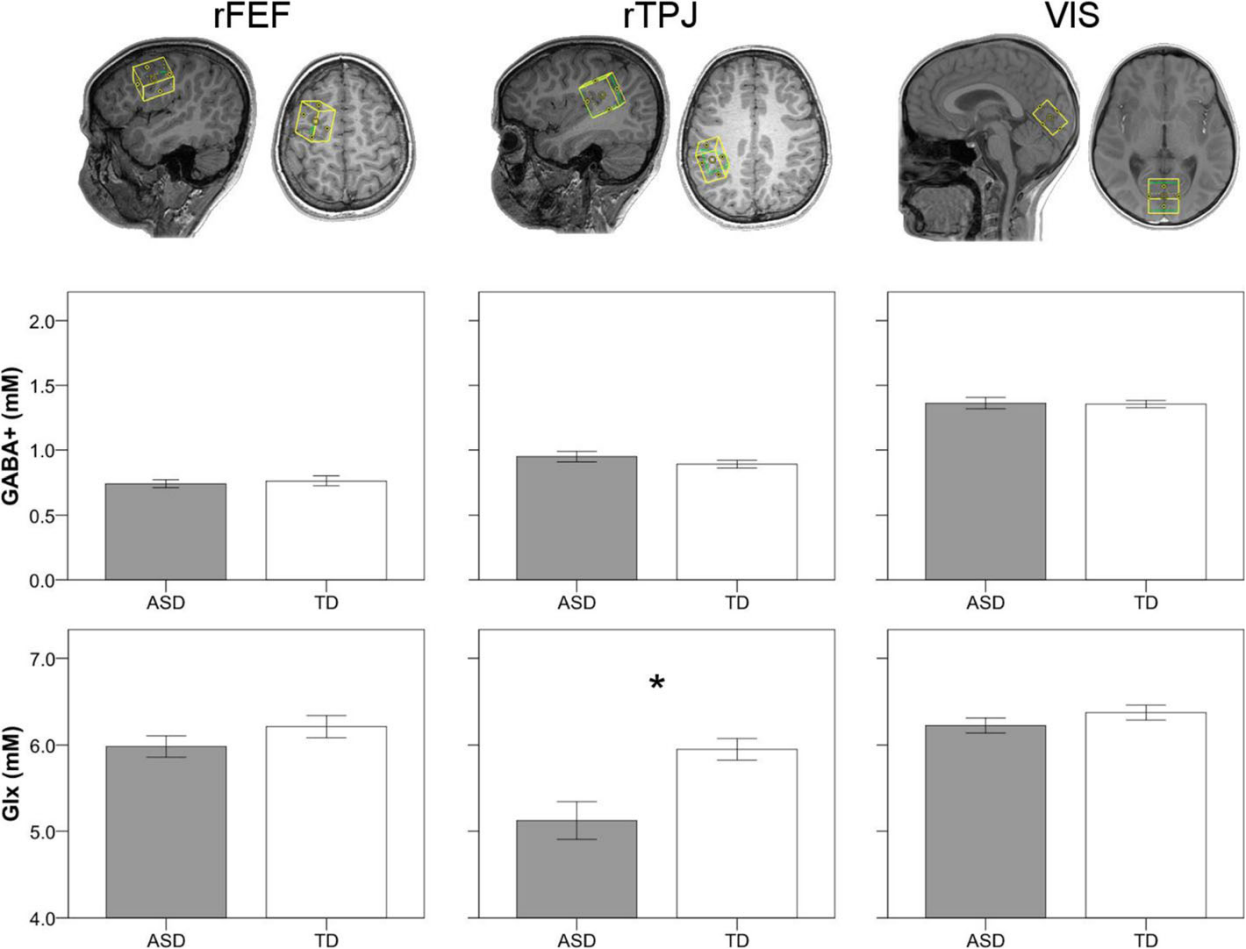
Volumes of interest (VOI) for right frontal eye fields (rFEF; left column), right temporal-parietal junction (rTPJ; center column), and visual cortex (VIS; right column) and non-transformed GABA+ and Glx values for ASD (gray) and TD (white) groups. Error bars represent ± 1 SEM. *p < .05

Volumes of interest (VOI) were placed in the right frontal eye fields (rFEF, 20 × 30 × 2), right temporal-parietal junction (rTPJ, 30 × 20 × 30), and visual cortex (VIS, 30 × 30 × 20) using anatomical landmarks (see [42], for more details; Fig. 2). Absolute quantification of GABA and Glx was performed on spectra from each VOI with LCModel V6.3-1B [45]. Basis sets used in LCModel were generated using density matrix simulation and using GABA coupling constants from Kaiser et al. [46]. Results from LCModel for each neurochemical were in mM and only neurochemicals with Cramer-Rao lower bounds (CRLB) of < 20% across all participants were used for subsequent analyses. Tissue segmentation to obtain percentages of white matter, gray matter, and cerebrospinal fluid (CSF) was performed using SPM12 (https://www.fil.ion.ucl.ac.uk/spm/). All metabolites were CSF-corrected with the exception of GABA, which was tissue-corrected using the method as described in Harris et al. [47] using an *α* = 0.5. Finally, MRS GABA+ and Glx values were log-transformed.

### Measures

#### Sensory Profile-2 (SP-2 [48])

The SP-2 is an 86-item caregiver-report questionnaire for 3- to 14-year-olds that measures four sensory processing categories: Seeking, Avoiding, Sensitivity, and Registration. Caregivers respond to each item using a five-point Likert scale with a score of 1 indicating a behavior that is present almost never and 5 indicating a behavior that is present almost always. The Seeking and Registration categories are related to hypo-sensitivity or high sensory thresholds, with Seeking being characteristic of a child that obtains sensory input and Registration being characteristic of a child that misses sensory input. The Sensitivity and Avoiding categories are related to hyper-sensitivity or low sensory thresholds, with Sensitivity associated with the degree that a child detects sensory input and Avoiding associated with the degree a child is bothered by sensory input. Higher scores in each quadrant are indicative of greater sensory symptoms.

## Results

### Alpha power

Absolute alpha power was entered into a 2 (group: ASD, TD) × 3 (ROI: frontal, central, posterior) mixed-model repeated measures ANOVA. There was a significant effect of ROI, *F*(2, 120) = 122.4, *p* < .001, η_p_^2^ = 0.67, as power was greater at the posterior compared to both frontal, *t*(61) = − 9.6, *p* < .001, and central, *t*(61) = − 13.5, *p* < .001, ROI, and greater at the frontal compared to central ROI, *t*(61) = 3.8, *p* < .001. Children with ASD showed marginally lower alpha power compared with TD children, *F*(1, 60) = 3.3, *p* = .075, η_p_^2^ = 0.05. Additionally, as illustrated in Fig. 1, there was a significant interaction between group and ROI, *F*(2, 120) = 5.0, *p* = .008, η_p_^2^ = 0.08. Follow-up *t* tests revealed significant group differences at the posterior ROI, *t*(60) = − 2.3, *p* = .024, but not the central, *t*(60) = − 1.4, *p* = .169, or frontal, *t*(60) = − 1.0, *p* = .307, ROIs. The percentage of epochs rejected did differ significantly between ASD (M = 33%; SD = 16%) and TD (M = 18%; SD = 15%) groups, *t*(60) = 3.7, *p* < .001; however, the percentage of rejected epochs was not associated with average alpha power across groups, *r*(60) = − .080, *p* = .536, and the pattern of results was unchanged when the percentage of rejected epochs was entered as a covariate in the ANOVA.

Lastly, a separate set of analyses was conducted to identify individual alpha frequency (IAF) using the method outlined by Corcoran and colleagues [49] and determine absolute low- (IAF-2Hz to IAF), high- (IAF to IAF+2Hz), and combined-alpha (IAF-2Hz to IAF+2Hz) power according to IAF. There were no differences in IAF between groups (ASD = 9.441 Hz; TD = 9.438 Hz, *p* = .99). Furthermore, the results for IAF-alpha power analyses were equivalent to our original analysis (i.e., significant interaction between group and ROI; significant difference at posterior ROI between ASD and TD).

### MRS

GABA+ and Glx values were entered into separate mixed-model repeated measures ANOVA with between-subjects factor group (ASD, TD) and within-subjects factor VOI (rFEF, rTPJ, and VIS). As previously reported in Edmondson et al. [42], while GABA+ levels varied by VOI, *F*(2, 54) = 152.7, *p* < .001, η_p_^2^ = 0.85, there were no significant between group differences, *F*(1, 27) = 0.01, *p* = .943, η_p_^2^ = 0.00, nor was there an interaction between group and VOI, *F*(2, 54) = 0.89, *p* = .416, η_p_^2^ = 0.03. Similar to GABA+, Glx varied significantly across VOI, *F*(2, 60) = 22.3, *p* < .001, η_p_^2^ = 0.43. However, in contrast to GABA+, Glx values were significantly lower in ASD, *F*(1, 30) = 9.2, *p* = .005, η_p_^2^ = 0.24. The significant main effect of group was subsumed by a significant group by VOI interaction, *F*(2, 60) = 6.00, *p* = .004, η_p_^2^ = 0.17. Follow-up *t* tests showed that the ASD group had significantly reduced Glx in the rTPJ, *t*(30) = − 3.2, *p* = .003, but not the rFEF, *t*(30) = − 1.3, *p* = .194, or VIS, *t*(30) = − 1.2, *p* = .233, VOI (see Fig. 2).

### Correlational analyses

#### GABA+, Glx, and alpha power

For all participants, increased VIS GABA+ was associated with greater frontal alpha power, *r*(60) = .408, *p* = .021. Within the TD group, VIS GABA+ values were significantly correlated with frontal alpha power, *r*(14) = .500, *p* = .048; however, these were not significantly correlated in the ASD group, *r*(14) = .362, *p* = .169. There were no other significant association between GABA+ values and alpha power across all participants or within ASD or TD groups. Additionally, there were no significant correlations between alpha power and Glx values across all participants, or within ASD and TD groups (all *p* values > .05; see Supplementary Table 2).

#### GABA+, Glx, alpha, and sensory processing

On the SP-2, the ASD and TD groups differed significantly on all quadrants (see Table 1). Across both groups, no significant correlations were found between SP-2 scores and alpha levels for any ROI (all *p* > .13). However, for the TD group greater central alpha power was associated with increased Sensitivity, *r*(29) = .398, *p* = .026, and Seeking, *r*(29) = .408, *p* = .023, scores. There were no significant associations between SP-2 scores and alpha power within the ASD group (all *p* > .49; see Supplementary Table 3).

**Fig. 3:**
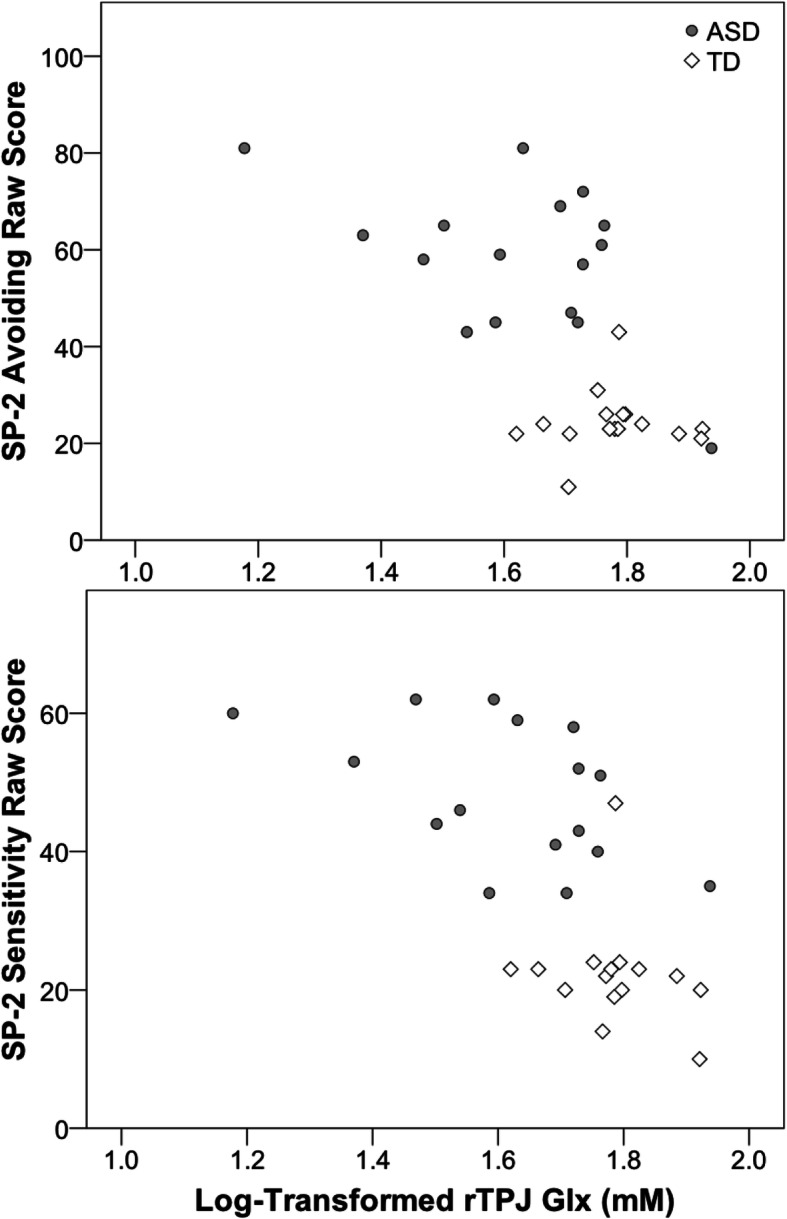
Scatterplots displaying associations between right temporal-parietal junction (rTPJ) Glx values and Avoiding and Sensitivity quadrant scores from the Sensory Profile-2 (SP-2)

For the MRS values, there was a significant association between rTPJ Glx values and Registration, *r*(30) = − .406, *p* = .021; Sensitivity, *r*(30) = − .581, *p* < .001; Avoiding, *r*(30) = − .619, *p* < .001; and Seeking, *r*(30) = − .447, *p* = .010, scores across all participants. These correlations were due, in part, to correlations within the ASD group, particularly for Sensitivity, *r*(14) = − .493, *p* = .052, and for Avoidance, *r*(14) = − .502, *p* = .048, scores (see Fig. 3). No significant correlations between MRS and SP-2 measures were present within the TD group (all *p* > .20; see Supplementary Table 4).

## Discussion

The present study sought to examine differences in resting-state alpha power between children with ASD and TD children as well as the associations between alpha levels, concentrations of excitatory and inhibitory neurotransmitters, and atypical sensory processing. To our knowledge this is the first study to examine whether atypical patterns of spontaneous brain activity, as measured by EEG, are associated with neurochemical differences in ASD. Consistent with a large body of previous research, children with ASD showed significantly increased sensory symptoms and reduced alpha power compared to their TD peers. However, inter-individual differences in alpha power within the ASD group were not associated with sensory processing differences, nor were they associated with region-specific concentrations of GABA or Glx. Children with ASD did show reduced Glx values in the right temporal-parietal junction compared to their TD peers, and atypically, decreased Glx was associated with elevated sensory processing symptoms in children with ASD. Together, these findings may provide insight into the neural underpinnings of sensory processing differences present in ASD.

### Alpha, excitation/inhibition, and sensory symptoms in ASD

In agreement with previous reports from infancy to adulthood (e.g., [16, 22]), we found reduced spontaneous absolute alpha power in children with ASD. Based on this atypical pattern of band-specific activity in ASD, Wang and colleagues [15] proposed that reduced mid-frequency (i.e., alpha) power may be related to an excitatory/inhibitory imbalance in ASD. However, our correlational analyses did not demonstrate any significant associations between reduced alpha levels and region-specific differences in the concentrations of either GABA+ or Glx in ASD. However, increased rFEF GABA+ concentrations were related to greater posterior alpha power across all participants and within the TD group specifically. Prior multi-method research has demonstrated that activation of frontal-parietal regions associated with attentional networks (including the rFEF) is related to resting and task-related changes in alpha power (e.g., [50–52]). These previous findings suggest that attention-related top-down signals may modulate the power of alpha-band activity. Additionally, inter-individual differences in top-down, goal-oriented control of behavior has been shown to be associated with region-specific GABA concentrations in the FEF [53]. Thus, while no differences were present in FEF GABA+ levels across groups, variable concentrations of inhibitory neurotransmitters within the FEF may contribute to individual differences in resting posterior alpha power within the TD (but not the ASD) group.

One other potential explanation for the absence of any correlations within the ASD may be that the regions examined in the present study do not include the generators of alpha activity that have previously been shown to contribute to differences present in ASD. For example, scalp-recorded alpha power may be associated with GABA-mediated thalamic activity [54, 55]. Prior research by Edgar and colleagues [56] reported significant correlations between thalamic volumes and visual alpha power for TD, but not ASD, children and suggested that the thalamus may contribute to alpha power differences in ASD. Additionally, fMRI evidence indicates that over-connectivity between the thalamus and cortex may be present in ASD [57, 58], and a recent multimodal fMRI-EEG study suggests that children with ASD with the largest reductions in alpha power may also tend to show greatest thalamo-cortical overconnectivity [59]. Thus, while there is limited evidence that GABA and Glx values within the thalamus may not differ in ASD [60–62], future work investigating the associations between neurochemical profiles and band-specific power differences may benefit from examination of thalamic contributions to atypical alpha activity in ASD.

Lastly, although we observed associations between alpha levels and Sensitivity and Seeking scores within the TD group, we did not find evidence of a relationship between alpha levels and sensory symptoms within children with ASD. Previous research demonstrating links between alpha and sensory symptoms has focused on frontal alpha asymmetry rather than absolute alpha power [27, 28]. Thus, hemispheric variations in alpha may be more sensitive to differences in sensory processing symptoms rather than absolute levels.

### GABA, glutamate, and sensory symptoms in ASD

As previously reported by Edmondson and colleagues [42], only Glx values in the rTPJ differed significantly in our sample of children with ASD, with no differences in GABA+ or Glx values present in any other VOI (see Edmondson et al. [42], for further discussion on the lack of between-group differences and discussion of other metabolites). Although Bernardi and colleagues [62] previously reported equivalent TPJ Glx levels, they did report reduced Glx values in the anterior cingulate cortex. Moreover, our finding of significantly lower Glx is consistent with several other studies that have documented reduced concentrations of Glx in ASD [63–66].

Contrary to prior research that has demonstrated associations between GABA levels and sensory processing in ASD [33–35], no associations were found between GABA+ values in any VOI and sensory processing symptoms across all participants or within TD or ASD groups. Nevertheless, our results are consistent with a recent study that found no association between GABA+ concentrations and behavioral and fMRI measures of visual spatial suppression [67]. Rather, we observed significant correlations between rTPJ Glx levels and all four sensory processing quadrants across both groups, which was primarily the result of associations within the ASD group, for Sensitivity and Avoidance subscales; greater scores on these subscales, indicative of increased hypersensitivity to sensory input, were associated with atypically decreased Glx levels in the ASD group, suggesting that reduced glutamate may contribute to sensory processing impairments in ASD.

Why are atypically decreased Glx values within the rTPJ associated with increased hyper-sensitivity in ASD? The rTPJ is a hub for social-cognitive processes and may coordinate communication about the information gathered from one’s external sensory environment with internal model-based predictions [68]. Further, the TPJ is involved in the processing of auditory, visual, and tactile sensory inputs (e.g., [69]) and is part of a larger brain network involved in the detection of novel multimodal stimuli [70, 71]. Processing of multisensory information may be disrupted in ASD [72], and others have hypothesized that deficits in multisensory integration may lead to a disorganized or chaotic perception of one’s sensory environment and sensory hyper-sensitivity [73]. Thus, neurochemical perturbations, specifically decreased glutamate within the rTPJ, may impact processing of multisensory information resulting in greater reactivity to sensory information.

In addition, a predictive coding account has also been used to explain differences in responding to sensory input in individuals with ASD (e.g., [74–77]). In particular, Lawson and colleagues [75] hypothesize that context-insensitive sensory drive and failures to optimize precision may result from atypically reduced glutamate in ASD. The mismatch-negativity (MMN) event-related potential (ERP) component, which is elicited by any perceivable change in a stream of repetitive sensory simulation, is thought to reflect early sensory prediction error processing [78]. In TD individuals, higher levels of Glx in the posterior superior temporal gyrus (which was also included in our TPJ VOI) are associated with faster MMN latency, suggesting that increased glutamate levels are related to more efficient prediction error signaling [79]. More recently, Kompus and colleagues [80] also demonstrated that increased levels of temporal Glx were associated with increased inter-regional functional connectivity between auditory cortex and the inferior parietal lobe, but not local connectivity within the temporal lobe, during unpredictable auditory stimulation, demonstrating the importance of Glx in facilitating long-range connectivity. Although the prior findings are mixed, a recent meta-analysis showed that smaller MMN amplitudes are consistently observed in individuals with ASD [81]. Importantly, atypically slower MMF (the magnetic equivalent to the MMN) latency [82] and smaller MMN amplitude [83] have been shown to be related to increased sensory sensitivity scores in ASD. Furthermore, Goris and colleagues [84] demonstrated that MMN amplitude was less modulated by global context in individuals with ASD, supporting predictive coding accounts of ASD. Thus, while it remains to be determined, disrupted glutamatergic signaling within the temporal-parietal cortex may contribute to previous reports of atypical MMN responses, and provide support for the hypoglutamatergic basis for the aberrant precision account of ASD.

### Limitations

Children and adolescents with ASD in the present study did provide significantly less usable EEG data; however, the amount of usable EEG was not associated with average alpha power across the two groups, nor did results change when percentage of usable data was included as a covariate. Additionally, because only a subset of participants completed the MRS portion of the study, our MRS analyses were limited to restricted number of participants. As such, correlations with MRS measures should be confirmed with a larger cohort of participants. Furthermore, correlational analyses were not corrected for multiple comparisons, and, thus, should be considered exploratory in nature. Finally, our interpretation of Glx values focuses on glutamate; however, Glx includes contributions from both glutamate and glutamine, and so should be interpreted with caution*.*

## Conclusions

Sensory processing impairments have a significant impact on the quality of life of individuals with ASD and their families. Thus, discovering the neurophysiological underpinnings of ASD symptomatology, including sensory processing differences, has the potential to provide insight into the heterogeneous nature of the ASD phenotype as well as to inform the identification of early diagnostic markers and development of novel intervention approaches. This study presents evidence that resting-state alpha levels are significantly reduced in children and adolescents with ASD compared to their TD peers, supporting a growing body of previous research. However, these differences were not associated inter-individual differences in sensory processing symptoms or region-specific variations in excitatory or inhibitory neurotransmitters. Instead, individuals with ASD exhibited reduced concentrations of Glx in the right temporal-parietal junction, and atypically decreased levels of Glx were associated with greater levels of sensory symptoms. These exploratory findings, which should be confirmed using a larger sample, suggest that a reduction in glutamatergic drive within the temporal-parietal cortex may contribute to hypersensitivity to sensory input in ASD.

## Supplementary Information

**Additional file 1.** Supplementary Tables

## Data Availability

The datasets used and/or analyzed in the current study are available from the corresponding author upon reasonable request.

## Abbreviations

ADOS: Autism Diagnostic Observation Schedule
ANOVA: Analysis of variance
ASD: Autism spectrum disorder
CSF: Cerebral-spinal fluid
DAS: Differential Abilities Scales, Second Edition
EEG: Electroencepholography
E/I: Excitatory/inhibitory
ERP: Event-related potential
fMRI: Functional magnetic resonance imaging
FEF: Frontal eye fields
GABA: Gamma-aminobutyric acid
Glx: Glutamate + glutamine
ICA: Independent component analysis
MMN: Mismatch negativity
MMF: Mismatch field
MRI: Magnetic resonance imaging
MRS: Magnetic resonance spectroscopy
ROI: Region of interest
SASICA: Semiautomatic selection of independent components for artifact
SCQ: Social Communication Questionnaire
SP-2: Sensory Profile, Second Edition
TD: Typically developing
TPJ: Temporal-parietal junction
VIS: Visual cortex
VOI: Volume of interest
WASI-II: Wechsler Abbreviated Scale of Intelligence. Second Edition
