# Supplementary material for: Associations between sensory processing and electrophysiological and neurochemical measures in children with ASD: an EEG-MRS study
Source: J Neurodev Disord. 2021 Jan 6;13:5. doi: 10.1186/s11689-020-09351-0 (PMC7788714; doi:10.1186/s11689-020-09351-0)
Supplement: Supplementary file 1 — Additional file 1. Supplementary Tables [file 11689_2020_9351_MOESM1_ESM.docx]

**Supplementary Materials**

**Results**

**Supplementary Table 1**

Participant Characteristics for MRS Sample

|  | ASD | TD | t-value | p-value |
| --- | --- | --- | --- | --- |
| N (male:female) | 16 (13:3) | 16 (12:4) | - | - |
| Age (years) | 11.9 (1.2);  10.5-14.6 | 11.1 (1.6);  8.9-15.0 | 1.61 | .12 |
| Verbal IQ | 104 (19);  75-154 | 109 (10);  97-127 | -0.82 | .42 |
| Nonverbal IQ | 106 (14);  85-132 | 110 (11);  94-132 | -0.97 | .34 |
| SP-2 Registration | 56 (16);  30-85 | 22 (8);  2-33 | 7.90 | < .001 |
| SP-2 Sensitivity | 48 (10);  34-62 | 21 (9);  0-47 | 7.98 | < .001 |
| SP-2 Avoiding | 58 (16);  19-81 | 24 (6);  11-43 | 7.95 | < .001 |
| SP-2 Seeking | 42 (13);  20-67 | 20 (8);  2-38 | 5.74 | < .001 |

**Supplementary Table 2**

Correlations between GABA and Glx Concentrations and Alpha Power

|  | | ASD (n = 16) | | | TD (n = 16) | | |
| --- | --- | --- | --- | --- | --- | --- | --- |
| FEF |  | Frontal | Central | Posterior | Frontal | Central | Posterior |
|  | GABA+ | .503 | .378 | .346 | -.295 | -.272 | -.412 |
|  | Glx | -.104 | .046 | .002 | -.076 | .167 | .159 |
| TPJ |  |  |  |  |  |  |  |
|  | GABA+ | .361 | .198 | .249 | -.146 | -.234 | -.447 |
|  | Glx | .065 | .082 | .299 | .090 | -.027 | -.169 |
| VIS |  |  |  |  |  |  |  |
|  | GABA+ | .362 | .027 | .141 | .500* | .364 | .162 |
|  | Glx | .157 | .206 | .240 | -.055 | -.126 | .226 |

**p* < .05

**Supplementary Table 3**

Correlations between Alpha Power and Sensory Profile Quadrants

|  | ASD (n = 31) | | | TD (n = 31) | | |
| --- | --- | --- | --- | --- | --- | --- |
|  | Frontal | Central | Posterior | Frontal | Central | Posterior |
| SP-2 Registration | 0.037 | 0.127 | 0.128 | 0.248 | 0.337 | -0.002 |
| SP-2 Sensitivity | -0.108 | 0.086 | 0.028 | 0.293 | 0.398* | 0.210 |
| SP-2 Avoiding | -0.117 | 0.034 | 0.080 | 0.156 | 0.236 | 0.015 |
| SP-2 Seeking | -0.087 | 0.044 | 0.079 | 0.273 | 0.408* | 0.259 |

**p* < .05

**Supplementary Table 4**

Correlations between GABA and Glx Concentrations and Sensory Profile Quadrants

|  | | ASD (n = 16) | | | | TD (n = 16) | | | |
| --- | --- | --- | --- | --- | --- | --- | --- | --- | --- |
| FEF |  | Registration | Sensitivity | Avoiding | Seeking | Registration | Sensitivity | Avoiding | Seeking |
|  | GABA+ | 0.238 | 0.067 | 0.481 | 0.130 | 0.069 | -0.057 | -0.096 | -0.077 |
|  | Glx | 0.024 | -0.070 | -0.142 | -0.116 | 0.223 | 0.174 | 0.059 | 0.324 |
| TPJ |  |  |  |  |  |  |  |  |  |
|  | GABA+ | 0.220 | -0.132 | -0.202 | -0.296 | -0.168 | -0.386 | -0.264 | -0.336 |
|  | Glx | 0.017 | -0.493 | -0.502* | -0.176 | 0.052 | -0.017 | 0.08 | 0.014 |
| VIS |  |  |  |  |  |  |  |  |  |
|  | GABA+ | 0.005 | -0.179 | -0.398 | -0.096 | -0.125 | -0.186 | -0.246 | -0.259 |
|  | Glx | -0.335 | 0.005 | -0.282 | -0.068 | 0.069 | 0.137 | 0.086 | 0.305 |

**p* < .05
